# AKI-CLIF-SOFA: a novel prognostic score for critically ill cirrhotic patients with acute kidney injury

**DOI:** 10.18632/aging.101161

**Published:** 2017-01-19

**Authors:** Dan-Qin Sun, Chen-Fei Zheng, Wen-Yue Liu, Sven Van Poucke, Zhi Mao, Ke-Qing Shi, Xiao-Dong Wang, Ji-Dong Wang, Ming-Hua Zheng

**Affiliations:** ^1^ Department of Nephrology, Affiliated Wuxi Second Hospital, Nanjing Medical University, Wuxi 214002, China; ^2^ Department of Nephrology, the First Affiliated Hospital of Wenzhou Medical University, Wenzhou 325000, China; ^3^ Department of Endocrinology, the First Affiliated Hospital of Wenzhou Medical University, Wenzhou 325000, China; ^4^ Department of Anesthesiology, Intensive Care, Emergency Medicine and Pain Therapy, Ziekenhuis Oost-Limburg, Genk, Belgium; ^5^ Department of Critical Care Medicine, Chinese People's Liberation Army General Hospital, Beijing, China; ^6^ Department of Hepatology, Liver Research Center, the First Affiliated Hospital of Wenzhou Medical University, Wenzhou 325000, China; ^7^ Institute of Hepatology, Wenzhou Medical University, Wenzhou 325000, China

**Keywords:** acute kidney injury, critically ill cirrhosis, prognosis, sequential organ failure assessment score

## Abstract

Critically ill cirrhotic patients with acute kidney injury (AKI) are associated with high mortality rates. The aims of this study were to develop a specific prognostic score for critically ill cirrhotic patients with AKI, the acute kidney injury - Chronic Liver Failure - Sequential Organ Failure- Assessment score (AKI-CLIF-SOFA) score. This study focused on 527 cirrhotic patients with AKI admitted to intensive care unit and constructed a new scoring system, the AKI-CLIF-SOFA, which can be used to prognostically assess mortality in these patient population. Parameters included in this model were analysed by cox regression. The area under the receiver operating characteristic curve (auROC) of AKI-CLIF-SOFA scoring system was 0.74 in 30 days, 0.74 in 90 days, 0.72 in 270 days and 0.72 in 365 days. Additionally, this study demonstrated that the new model had more discriminatory power than chronic liver failure- sequential organ failure assessment score (CLIF-SOFA), SOFA, model for end stage liver disease (MELD), kidney disease improving global outcomes (KDIGO) and simplified acute physiology score II (SAPS II) (auROC: 0.72, 0.66, 0.64, 0.62, 0.63 and 0.65 respectively, all P < 0.05) for the prediction of the 365-days mortality. Therefore, AKI-CLIF-SOFA demonstrated a valuable discriminative ability compared with KDIGO, CLIF-SOFA, MELD, SAPS II and SOFA in critically ill cirrhotic patients with AKI.

## INTRODUCTION

Acute kidney injury (AKI) is a severe complication in critically ill cirrhotic patients, which occurs in up to 50% of the patients admitted with cirrhosis [1]. The most important causes of AKI are related with the development of severe complications of cirrhosis, such as spontaneous bacterial peritonitis [2], hepatorenal syndrome [3], variceal bleeding [4] and the majority are common reasons for admission to an intensive care unit (ICU). As universally accepted, AKI is a strong predictor for mortality in patients with critically ill cirrhosis [5].

In recent years, two separate bodies developed and published two consensus definitions for AKI: the Acute Dialysis Quality Initiative group for the Risk, Injury, Failure, Loss of Renal Function and End-Stage Renal Disease (RIFLE) criteria; and the Acute Kidney Injury Network (AKIN) group for the AKIN criteria [6, 7]. In 2012, the Kidney Disease Improving Global Outcomes (KDIGO) criteria, were created based on the RIFLE and AKIN classifications for prediction of hospital mortality [8]. The KDIGO criteria have been validated by many investigations for patients with AKI [9-11]. Until now, there are many established liver-specific and general ICU prognostic models. The Chronic liver Failure-Sequential Organ Failure Assessment (CLIF-SOFA) score is an excellent prognostic evaluation tool derived from the widely used sequential organ failure assessment score (SOFA) score for intensive care unit patients, aiming for a better reflection of the impact of organ failures in the context of cirrhosis [12]. The higher the CLIF-SOFA score, the higher the mortality rate in patients with acute-on-chronic liver failure [13]. Models for End-Stage Liver Disease (MELD) are also widely utilized for evaluating the severity of critically ill cirrhosis [14]. The Simplified acute physiology score (SAPS II) and SOFA score are widely used scoring systems used to assess the prognosis at ICU [15]. However, there is a lack of a specific prognostic score focusing on critically ill cirrhotic patients with AKI.

In this study, the main object was therefore, to develop a new score for critically ill cirrhotic patients with AKI in order to improve the predicting accuracy of the CLIF-SOFA score for hospital mortality. In addition, the study compared the performance of the novel score with CLIF-SOFA, MELD, SAPS II, SOFA and KDIGO.

## RESULTS

### Baseline characteristics of acute kidney injury in critically ill cirrhotic patients

From June 2001 to October 2012, 527 critically cirrhosis ill patients with AKI met our criteria and were included in our study. The mean age of these patients was 57 years; 362 of the patients were male (68.7%). The in-hospital mortality rates were observed: for 30 days, 45.7%; for 90 days, 56.5%; for 270 days, 63.4% and for 365 days, 64.3%. Table 1 lists the patient demographic data, clinical characteristics laboratory parameters and clinical scores of both survivors and non-survivors. This study revealed that the demographic data were almost similar and the most frequent ethnic group was Caucasians in two groups. Compared with patients in the survival group, non-survivors were slightly older and had significantly higher temperatures, systolic blood pressure (SBP), diastolic blood pressure (DBP), mean arterial pressure (MAP), glucose, potassium, blood urea nitrogen (BUN), partial pressure of oxygen (PaO_2_), creatinine, lactate, bilirubin and urine output. Moreover, the clinical scores were also significantly different between survival and non-survival group.

**Table 1:** Characteristics of critically Ill cirrhosis patients with acute kidney disease on the first day of admission, stratified by survival

Variable	Survivors (n=188)	Non-survivors (n=339)	P-value
**Demographic parameters**				
Age, year	56.1 ± 10.4	58.8 ± 12.2	0.010
Sex, male no. (%)	128 (68.1%)	234 (69.0%)	NS (0.824)
Height, cm	172.2 ± 9.8	171.7 ± 9.9	NS (0.619)
Weight, kg	82.3 ± 20.5	84.4 ± 21.2	NS (0.291)
**Survival time**				
Death time after admission	365.0 ± 0.0	37.23 ± 60.1	< 0.001
**Ethnicity**				
White no. (%)	143 (76.1%)	227 (67.0%)	0.015
African black no. (%)	14 (7.4%)	24 (7.4%)	
Other no. (%)	31 (16.5%)	88 (26.0%)	
**Clinical parameters**				
Heart rate, n. (%)	89.9 ± 19.6	90.8 ± 19.8	NS (0.584)
Respiratory rate,	40.5 ± 33.3	40.3 ± 32.9	NS (0.971)
Temperature,°C	36.6 ± 0.8	36.3 ± 1.1	0.003
SBP, mmHg	117.0 ± 22.1	110.9 ± 21.8	0.002
DBP, mmHg	62.6 ± 16.4	57.7 ± 14.8	< 0.001
MAP, mmHg	80.8 ± 16.8	75.4 ± 15.1	< 0.001
Vasopressin used, n. (%)	82 (43.6%)	221 (65.2%)	< 0.001
**Laboratory parameters**				
Glucose, mg/dL	142.3 ± 75.7	127.7 ± 57.7	0.013
White blood cell, 10^9^/L	11.3 ± 6.7	12.0 ± 7.8	NS (0.282)
Platelet,10^9^/L	134.9 ± 105.1	130.5 ± 92.2	NS (0.618)
Sodium, mEq/L	134.4 ± 7.3	134.3 ± 7.1	NS (0.801)
Potassium, mEq/L	4.2 ± 0.9	4.4 ± 1.0	0.012
BUN, mg/dL	39.8 ± 28.0	47.1 ± 29.9	0.006
PO_2_, mmHg	166.9 ± 123.5	127.7 ± 103.8	< 0.001
PCO_2_, mmHg	37.7 ± 9.9	37.4 ± 11.4	NS (0.754)
FIO_2_	56.8 ± 33.4	66.4 ± 32.1	0.001
Bicarbonate, mEq/L	21.1 ± 5.1	20.7 ± 5.6	NS (0.339)
Creatinine, mg/dL	2.0 ± 1.4	2.3 ± 1.8	0.045
Creatinine (24h), mg/dL	1.8 ± 1.2	2.4 ± 1.8	< 0.001
Lactate, mg/dL	2.9 ± 2.2	3.9 ± 3.3	0.001
INR	1.9 ± 0.8	2.4 ± 3.6	0.053
Bilirubin, mg/dL	6.5 ± 9.3	9.9 ± 11.2	< 0.001
Urine output, ml	1837.8 ± 2167.5	997.2 ± 1583.1	< 0.001
**Clinical scores**			
CLIF-SOFA	9.7 ± 3.4	11.8 ± 3.6	< 0.001
MELD	21.9 ± 9.3	26.3 ± 10.9	< 0.001
SAPSII	43.2 ± 15.0	51.3 ± 14.2	< 0.001
SOFA	8.3 ± 3.5	10.1 ± 3.6	< 0.001
KDIGO	2.0 ± 0.9	2.5 ± 0.8	< 0.001
AKI-CLIF-SOFA	1.7 ± 1.1	2.6 ± 1.1	< 0.001

NOTE: CLIF-SOFA: chronic liver failure - sequential organ failure assessment score; DBP: diastolic blood pressure; INR: international normalized ratio; MELD: model for end-stage liver disease; AKI-CLIF-SOFA: acute kidney disease-chronic liver failure - sequential organ failure assessment score; SBP: systolic blood pressure; MAP: mean arterial pressure; KDIGO: kidney disease improving global outcomes; SAPSII: simplified acute physiology score; BUN: blood urea nitrogen; PaO_2_: partial pressure of oxygen; PCO_2_: partial pressure of carbon dioxide; FIO_2_: fraction of inspiration O_2_; NS: not significance.

### Development and construction of AKI-CLIF-SOFA score

**Figure 1:**
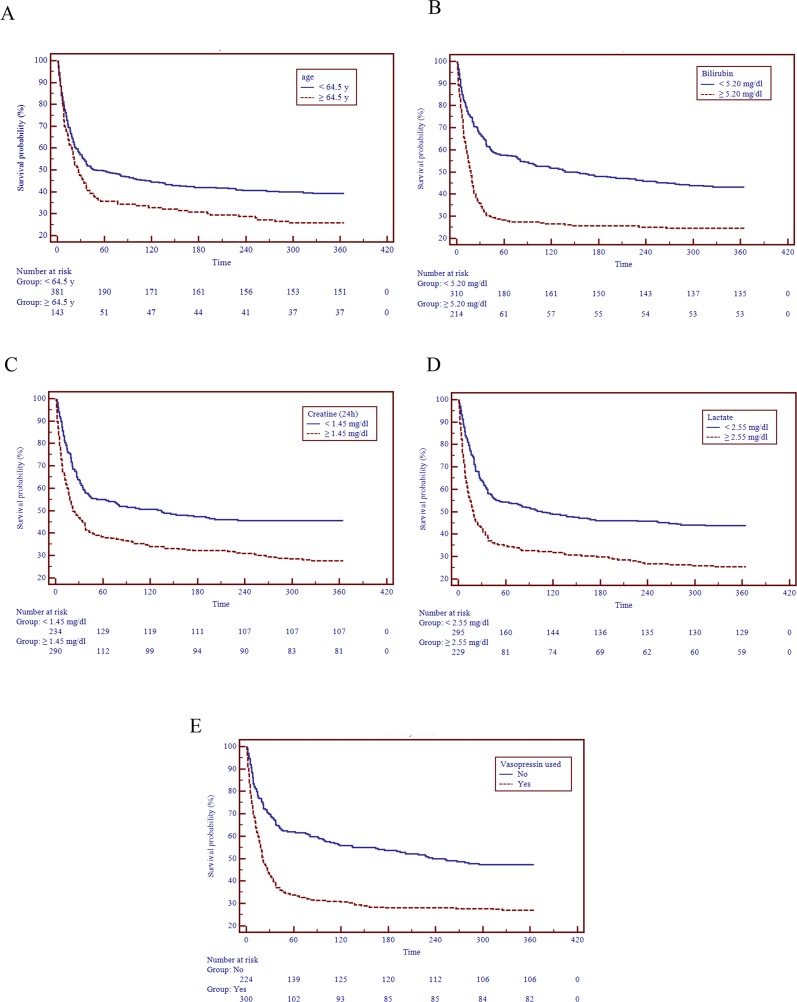
Survival distributions of different risk lev els of the AKI-CLIF-SOFA scoring system

To identify predictors of mortality of critically ill cirrhotic patients with AKI, the AKI-CLIF-SOFA scorings system was developed. Therefore, univariate and multivariate analyses for the study end-points were used to compute clinical and laboratory characteristics at patients’ enrollment (Table 2). Multivariate analysis demonstrated that age (HR 1.02, 95%CI 1.01-1.04), bilirubin (HR 1.03, 95%CI 1.02-1.04), 24h creatinine (HR 1.15, 95%CI 1.08-1.22), lactate (HR 1.10, 95%CI 1.06-1.13), vasopressin used (HR 1.68, 95%CI 1.32-2.13) were identified as independent risk factors for the mortality of these patients. Finally, these five parameters were included in novel scoring system: AKI-CLIF-SOFA. In addition, five optimal cutoff points were selected to distinguish two categorical variables that were directly associated with an increased mortality risk (Table 3). Subgroup analysis demonst-rated that patients with age ≥ 64.5y, bilirubin ≥ 5.2 mg/dl, 24h creatinine ≥ 1.45 mg/dl, lactate ≥ 2.55 mg/dl and vasopressin used had a poorer survival probability (Figure 1).

**Table 2:** Univariate and multivariate analysis of the association between mortality and clinical and laboratory characteristics in patients

	Univariate analysis			Multivariate analysis		
Variables		HR		95%CI		P		HR		95%CI		P	
**Age**		1.01	1.00-1.02	0.008	1.02	1.01-1.04	<0.001
**Bilirubin**		1.02	1.01-1.03	<0.001	1.03	1.02-1.04	<0.001
**Creatinine (24h)**		1.19	1.12-1.26	<0.001	1.15	1.08-1.22	<0.001
**Lactate**		1.10	1.06-1.34	<0.001	1.10	1.06-1.13	<0.001
Vasopressin used*	1.90	1.52-2.38	<0.001	1.68	1.32-2.13	<0.001

Note: CI: confidence interval; HR: hazard ratio

* Dichotomous values

**Table 3:** Variables of acute kidney disease-chronic liver failure - sequential organ failure assessment score

Variables	0	1
**Creatinine (24h)**		< 1.45 mg/dL	≥ 1.45 mg/dL
**Bilirubin**		< 5.20 mg/dL	≥ 5.20 mg/dL
**Age**		< 64.5 y	≥ 64.5 y
**Lactate**		< 2.55 mg/dL	≥ 2.55 mg/dL
**Vasopressin used**		no	yes

**Figure 2:**
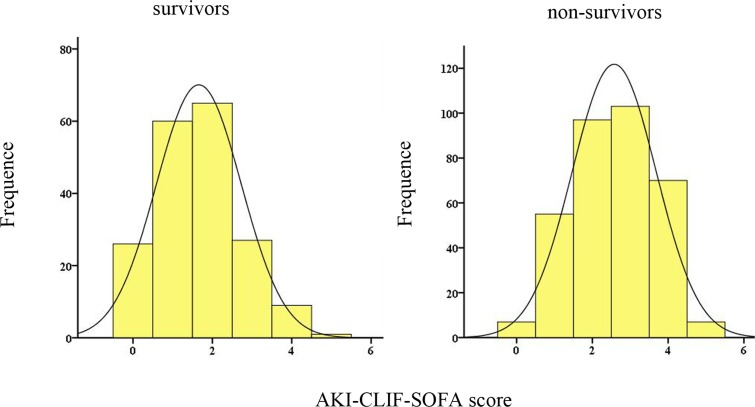
Distribution of the AKI-CLIF-SOFA score among survivors and non-survivors

**Figure 3:**
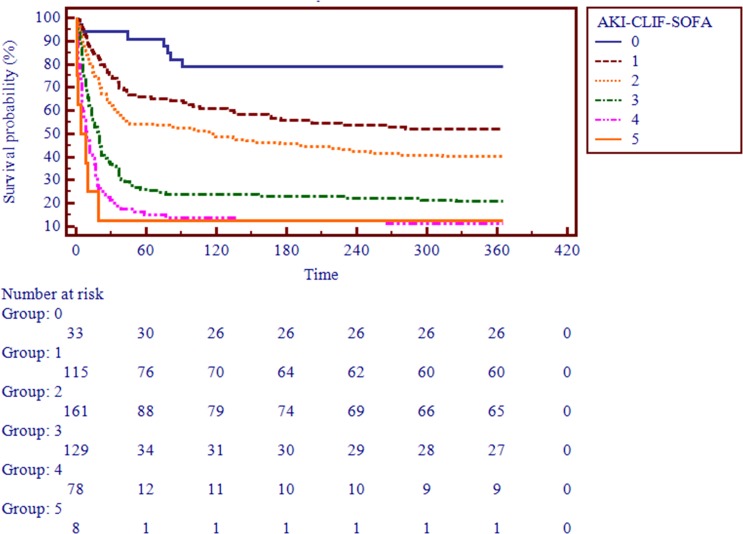
Survival probability based on the AKI-CLIF-SOFA score

After applying the AKI-CLIF-SOFA score for enrolled subjects, the minimum and maximum values were 0 and 5. In our study, the mean score of non-survivors and survivors were 2.5 ± 0.8, 2.0 ± 0.9, respectively. Moreover, the distribution of the novel score was showed in the Figure 2. Figure 3 demonstrated that a progressive and significant increase in the mortality rate was observed which correlated with the increasing AKI-CLIF-SOFA score.

**Figure 4:**
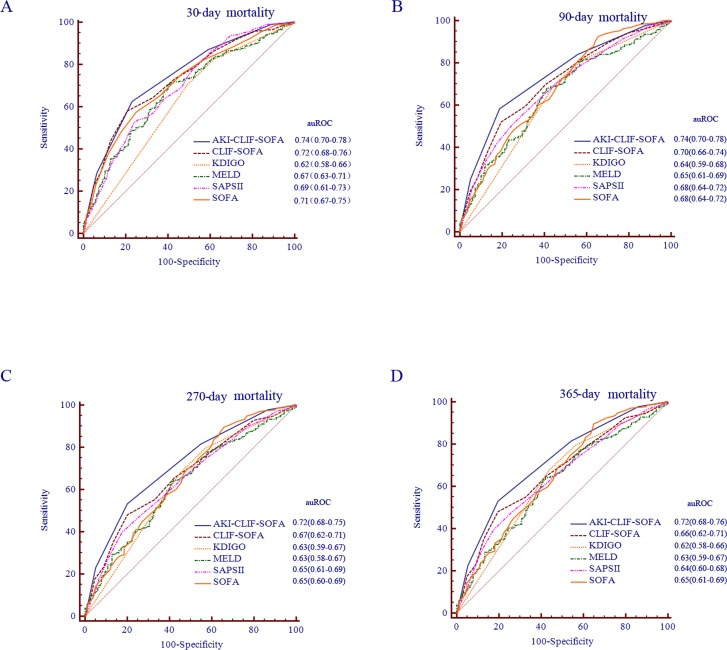
ROC analysis of the prognostic efficiency of AKI-CLFI-SO FA score and other models at different time periods

The performance of AKI-CLIF-SOFA to predict the mortality was presented in Table 4 and figure 4. The the area under the receiver operating characteristic curve (auROC) of new scoring system were 0.74 (95% CI: 0.70-0.78) for 30 days, 0.74 ((95% CI: 0.70-0.78) for 90 days, 0.72 (95% CI: 0.68-0.75) for 270 days and 0.72 (95% CI: 0.68-0.76) for 365 days analysis. Moreover, we used an optimal cutoff point of 2 for the 365-days mortality according to best Youden index. The associated sensitivities and the specificities were 53.1% and 80.32% respectively.

### Comparison of discrimination for predicting in-hospital mortality according to AKI-CLIF-SOFA, CLIF-SOFA, SOFA, MELD, SAPS II and KDIGO scores

The ability to predict mortality of different scores for critically ill cirrhotic patients with AKI was illustrated by the auROC for the different scores: CLIF-SOFA 0.66, MELD 0.62, SAPS II 0.65, KDIGO 0.63 and SOFA 0.64. The optimal cutoff point according to best Youden index for each score, and corresponding sensitivity, specificity, PPV, NPV, LR+ and LR- are shown in table 4. Furthermore, the analysis carried out by comparing the auROCs corresponding to AKI-CLIF-SOFA, CLIF-SOFA, MELD, SAPS II, KDIGO and SOFA for 30-days, 90-days, 270-days and 365-days mortality all confirmed the superiority of AKI-CLIF-SOFA and the improvement in predictive ability with respect to the other scores.

## DISCUSSION

To our knowledge, many studies investigated the KDIGO criterionin critically ill patients [16-19]. Moreover, the International Club of Ascites (ICA) proposed and modified an adaptation of the KDIGO criteria to define AKI in patients with cirrhosis [20-22]. Nevertheless, only one study had generated a novel prognostic scoring system for renal-specific scores from critically ill patients with cirrhosis and this have not been widely endorsed [23]. The new score (MBRS score: MAP + bilirubin + respiratory failure + sepsis) was derived from 111 cirrhotic patients with acute renal failure. However, in this study, the definition of acute kidney failure was based on the RIFLE classification and the modification of diet in renal disease (MDRD) formula was applied to estimate baseline serum creatinine (SCr) concentrations. As we know, the MDRD formula is inaccurate in the estimation of estimation of glomerular filtration rate (GFR) in cirrhotic patients. In addition, the new score guaranteed a strait forward use and provided prognostic information using variables that are easily available for clinicians who first encountered with the patients on the day of admission to the hospital.

The study, which included 527 critically ill cirrhotic patients with AKI, demonstrated that an increasing trend of hospital mortality with progression of the new AKI-CLIF-SOFA score. The score performed better than the established and commonly used acute physiology, renal-specific and liver-specific scores in our cohort.

In the current AKI-CLIF-SOFA score, parameters consist of 24h creatinine, bilirubin, age, lactate and vasopressin used (0 or 1 for each variable, range 0-5 points). It was important to note that bilirubin and creatinine played an important role in predicting mortality as was also observed in the CLIF-SOFA score. As is generally accepted, creatinine is the most practical biomarker of renal function in patients. Furthermore, after adjusting for other parameters, the prognosis of creatinine at 24 hours after admission was superior to creatinine on admission in analysis. Elevated bilirubin concentration has been shown to be associated with mortality of patients with liver-disease. Age, lactate, and vasopressin used added discriminative power as organ function predictors, explaining the higher accuracy of AKI-CLIF-SOFA with respect to other renal scores. Vasopressin used is also an important indicator of systemic derangements related to circulatory failure [24]. High lactate levels are also considered essential related with aggravating events, such as sepsis, respiratory, or cardiac failure [25, 26].

To compare the performance of this score against the current gold standards, KDIGO, CLIF-SOFA, MELD, SOFA and SAPSII score, we used the auROC analysis. In several recent studies, CLIF-SOFA demonstrated a favorable performance and proved to be a strong predictor for mortality in patients with cirrhosis [27-29]. Nevertheless, the CLIF-SOFA score had an inappropriate discriminatory power for predicting in-hospital mortality in these special patients (auROC = 0.66). The auROC analysis clearly showed that AKI-CLIF-SOFA was significantly more accurate in predicting 30-days, 90-days, 270-days and 365-days mortality than other scores. Therefore, the new scoring system provided a better discriminative ability than other liver-specific and kidney-specific scores. Moreover, the Kaplan-Meier survival curve showed that higher AKI-CLIF-SOFA score groups were associated with a higher risk for hospital mortality. However, validation of our model should be undertaken to confirm its clinical utility.

A few potential limitations need consideration. Firstly, because our study population was exclusively included from a single center, a potential selection bias might exist which limits the generalization of our findings. Secondly, sequential measurement of these scoring systems may reflect the dynamic aspects of clinical diseases, thus providing superior information on mortality risk. Thirdly, multi-center large-scale studies at more than 365-days follow-up are needed to further verify its prognosis of our new scoring system. Fourth, in order to use this scoring system as a model, predictive analytics request a validation of the model in order the model is suited for other patients then those included in this manuscript. Therefore cross-validation or bootstrap validation could be useful.

In conclusion, this is the largest study to evaluate prognostic scoring system for critically ill cirrhotic patients with AKI. The AKI-CLIF-SOFA scoring system has the best discriminatory power for predicting in-hospital mortality in study cohorts and may be an optimal scoring system for critically ill cirrhosis with AKI. Further research is needed to clarify the validity of AKI-CLIF-SOFA score.

## MATERIALS AND METHODS

### The database

The Multi-parameter Intelligent Monitoring in Intensive Care III version 3.0 (MIMIC-III v3.0) database is a large, single-center database comprising information relating to patients admitted to critical care units [30]. The database included general information (patient demographics, hospital admission and discharge dates), vital signs, medication, laboratory tests, fluid balance and notes and reports. The establishment of the database was approved by the Institutional Review Boards of the Beth Israel Deaconess Medical Center (Boston, MA) and the Massachusetts Institute of Technology (Cambrige, MA, USA). Currently, the database consisted of more than 40,000 ICU patients admitted to Beth Israel Deaconess Medical Center from June 2001 to October 2012. Our permission to access the database was approved after competition of the NIH web based training course named “Protecting Human Research Participants” (Our certification number: 1605699).

In this study, we included 527 consecutive patients with cirrhosis admitted to ICU, complicated with AKI and were followed-up for 365-days. Reasons for exclusion were: pediatric patients (age 18 years or below), patients admitted to the hospital for < 24h, patients with previous end-stage renal disease and received regular RRT, and patients with a history of liver transplantation.

### Definition

Liver cirrhosis was defined when at least two of the following criteria were satisfied: 1) ultrasonographic evidence of a small-sized liver with and without splenomegaly/ascites; 2) hypoalbuminemia (serum albumin < 35 g/L); 3) aminotransferase to platelet ratio (× 10^9^/L) × 100 > 2. Alcoholic cirrhosis of the liver was considered with a daily alcohol consumption of more than 80 g/day for at least five years.

The occurrence of AKI was determined based on the KDIGO classification. The definition is that SCr changes ≥ 1.5* baseline to have occurred within the prior 7 days or a 0.3 mg/d increase in SCr must occur within a 48 hours period or Urine output < 0.5 ml/kg/h * 6 hours.

1. Stage 1: Increase in SCr ≥ 1.5* baseline or of 0.3 mg/dl or Urine output < 0.5 ml/kg/h * 6 hours
2. Stage 2: Increase in SCr ≥ 2.0* baseline or Urine output < 0.5 ml/kg/h * 12 hours
3. Stage 3: Increase in SCr ≥ 3.0* baseline or increase in serum creatinine to ≥ 4.0 mg/dl or initiation of RRT or Urine output < 0.5 ml/kg/h * 12 hours

For patients without an available SCr value prior to hospitalization, we followed the recommendations of ICA and used the first SCr value measured during hospitalization as the baseline SCr [32]. Urine output was observed for the first 24h after ICU admission and was corrected for body weight.

### Data collection

Our investigators extracted demographic parameters, survival time, clinical parameters and laboratory parameters. The clinical parameters, which included heart rate, respiration, temperature, SBP, DBP, and MAP, were derived by ICU nurses from the hospital's on-line information systems. The laboratory parameters from routine tests on admission, including glucose, white blood cell, platelet, sodium, potassium, BUN, PaO_2_, partial pressure of carbon dioxide (PCO_2_), fraction of inspiration O_2_ (FIO_2_), bicarbonate, lactate, international normalized ratio (INR) and bilirubin were organized into a relational database. Additionally, the urine output was measured for the first 24h after ICU admission and recorded at least 6h. SCr was measured when clinically needed, at least once in 24 hours. The other data included age, sex, height, weight, ethnicity, vasopressin used, renal replacement therapy (RRT) used and survival time. Mortality data were censored after hospital discharge and were obtained by Social Security Death Records from the United States government. For all patients the CLIF-SOFA, MELD, SAPS II, KDIGO and SOFA were calculated. The start date was the date of patient's admission and the primary end points were defined at 30-days, 90-days, 270-days and 365-days for all-cause mortality.

Data extraction was performed using Oracle SQL Developer version 3.0 (Oracle Corporation, Redwood Shores, CA). Because this study is retrospective, no ethical approval was required for these analyses of non-patient identifiable and anonymous data.

### Statistical analysis

Data were presented as mean and standard derivations for continuous and normally distributed variables, or frequencies (percentage) for categorical variables. The Kolmogorov–Smirnov test was calculated for assessing the distribution of the variables. For comparisons, the Student's t -test and the Mann-Whitney test was used for continuous baseline characteristics of the each group for continuous variables with or without normal distribution, respectively. The Chi-square test was performed for categorical variables. Cox regression was used for univariate and multivariate analysis. Survival curves were constructed based on Kaplan-Meier estimates and comparisons were performed using the log-rank test.

Discrimination was examined using auROC. All the patients were enrolled for a comparison of the discriminative value of the CLIF-SOFA, MELD, SAPS II, KDIGO and SOFA scores, as well as for the AKI-CLIF-SOFA score for predicting mortality risks at 30-days, 90-days, 270-days and 365-days. The optimal cut-off point was identified based on the maximal Youden index (sensitivity + specificity − 1). In addition, the corresponding sensitivity, specificity, positive likelihood ratio (PLR), negative likelihood ratio (NLR), positive predictive value (PPV), negative predictive value (NPV) were calculated according to the auROC results. Statistical analyses were performed using SPSS version 18.0 software (IBM, Armonk, NY), MedCalc version 12.7 (MedCalc Software, Ostend, Belgium).

## Abbreviations

CLIF-SOFA: chronic liver failure - sequential organ failure assessment score
DBP: diastolic blood pressure
INR: international normalized ratio
MELD: model for end-stage liver disease
AKI-CLIF-SOFA: acute kidney disease-chronic liver failure - sequential organ failure assessment score
SBP: systolic blood pressure
MAP: mean arterial pressure
KDIGO: kidney disease improving global outcomes
SAPS II: simplified acute physiology score
BUN: blood urea nitrogen
PaO_2_: partial pressure of oxygen
PCO_2_: partial pressure of carbon dioxide
FIO_2_: fraction of inspiration O_2_
NS: not significance
AKI: acute kidney injury
auROC: area under the receiver operating characteristic curve
ICU: intensive care unit
KDIGO: the Kidney Disease Improving Global Outcomes
MDRD: modification of diet in renal disease
SCr: serum creatinine
